# Complement System and Alarmin HMGB1 Crosstalk: For Better or Worse

**DOI:** 10.3389/fimmu.2022.869720

**Published:** 2022-04-28

**Authors:** Christine Gaboriaud, Marie Lorvellec, Véronique Rossi, Chantal Dumestre-Pérard, Nicole M. Thielens

**Affiliations:** ^1^Univ. Grenoble Alpes, CEA, CNRS, IBS, Grenoble, France; ^2^Laboratoire d’Immunologie, Pôle de Biologie, CHU Grenoble Alpes, Grenoble, France

**Keywords:** complement system, interplay, HMGB1, auto-immunity, lupus, inflammation, periodontitis

## Abstract

Our immune system responds to infectious (PAMPs) and tissue damage (DAMPs) signals. The complement system and alarmin High-Mobility Group Box 1 (HMGB1) are two powerful soluble actors of human host defense and immune surveillance. These systems involve molecular cascades and amplification loops for their signaling or activation. Initially activated as alarm raising systems, their function can be finally switched towards inflammation resolution, where they sustain immune maturation and orchestrate repair mechanisms, opening the way back to homeostasis. However, when getting out of control, these defense systems can become deleterious and trigger serious cellular and tissue damage. Therefore, they can be considered as double-edged swords. The close interaction between the complement and HMGB1 pathways is described here, as well as their traditional and non-canonical roles, their functioning at different locations and their independent and collective impact in different systems both in health and disease. Starting from these systems and interplay at the molecular level (when elucidated), we then provide disease examples to better illustrate the signs and consequences of their roles and interaction, highlighting their importance and possible vicious circles in alarm raising and inflammation, both individually or in combination. Although this integrated view may open new therapeutic strategies, future challenges have to be faced because of the remaining unknowns regarding the molecular mechanisms underlying the fragile molecular balance which can drift towards disease or return to homeostasis, as briefly discussed at the end.

## 1 Introduction: Complement C1, C3 and HMGB1 are Constitutive Multifunctional Components

Several physiological processes keep us healthy, and are modulated throughout the human life, with major variations occurring at the early and late (>65y) stages. Some processes may act silently and get discovered only when they malfunction or drift towards a disequilibrium inducing disease. This idea will be illustrated through two examples of important molecular immune players which are constitutively expressed. This review will mainly focus on the C1 and C3 components of the complement system, a major front line in the host defense, and on the nuclear protein HMGB1, which functions as an alarmin in the extracellular space. Interestingly, these molecules are always present, except in some very rare cases of deficiency. HMGB1 is strongly conserved through evolution (99 % sequence identity within mammals), and it is lethal when completely missing. Complement C1s and C1q sequence alterations are also quite rare. The classical function of these proteins has been elucidated independently, and will only be briefly summed up, since this review will focus on possible molecular crosstalk that has recently come to light.

We will first shortly describe how alarm signals are raised and amplified through these systems independently, and possibly amplified or down-regulated by their crosstalk. Alert signals are raised by the detection of molecular motifs (associated molecular patterns, AMPs) associated with infectious (pathogens, PAMPs), tissue damage (DAMPS) or cell death (apoptotic cell, ACAMPs) context. Among similarities between the two systems, we aim to illustrate amplification mechanisms as well as the plethora of receptors and signaling pathways involved, their effect thus depending on the local tissues and environment.

**Figure 1:**
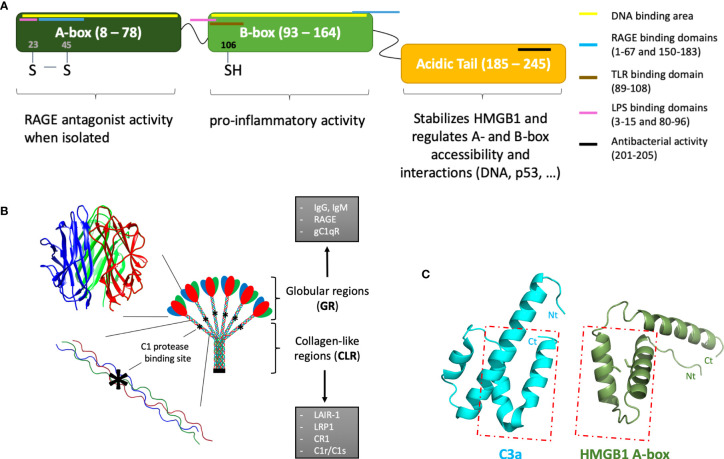
Introductory molecular schemes on HMGB1, C1q, and C3a underlying their multiple functions. **(A)** HMGB1 function is modulated by its two DNA-binding boxes (A-box and B-box) and an acidic C-terminal tail. By binding to different receptors, targets and partners (see color bars), HMGB1 can play multiple extracellular functions. Interactions restricted to intracellular functions are not shown. The X-ray structure of the A-box is shown in **(C)**, with its two cysteines shown as sticks. HMGB1 location within cells depends on its nuclear localization signals (NLS), located in the A-box (24–44) and B-box (179–185). **(B)** The largest multivalent C1q molecule can bridge different receptors or partner molecules through two regions with strongly different structural shapes and interaction properties. C1q is assembled from three different chains (red, blue, green), with numerous post-translational modifications, especially in the collagen-like regions (CLR). The globular regions (GRs) are mostly involved in the multivalent binding of target surfaces. Its main target ligands are surfaces opsonized by IgG, IgM, CRP, PTX3. C1q GR can also directly bind to DNA, phosphatidylserine, annexins, GAPDH, as well as to lipopolysaccharides (LPS) or virus surface proteins (HIV-1 gp41, HTLV-1 gp21). Some surface receptors bind to C1q GR, such as RAGE, gC1qR, DC-SIGN or Siglec 3 (CD33). C1q CLR mostly binds effector molecules, such as the C1r and C1s proteases, or LAIR-1, CR1, LRP1 surface receptors. These latter interactions are almost exclusive, because the proteases compete with receptor binding. The C1r/C1s protease binding site (*) has been finely investigated by C1q site-directed mutagenesis (1). **(C)** The similarity between HMGB1 A-box and C3a is very limited, despite the fact that both are small helical domains which can bind RAGE. The C3a and C5a anaphylatoxins are the smallest fragments released upon C3 and C5 cleavage. They are composed of helices, which is a feature common to HMGB1 boxes. However, only the two C-terminal helices of the A-box can roughly be superimposed to the two N-terminal helices of C3a (see box), in the opposite direction: therefore, the similarity between the two molecules is very limited [PDB codes 1ckt (2) and 4hw5 (3) for A-box and C3a, respectively].

The modular assembly of the smallest (HMGB1) and largest (C1q) proteins in the focus of this review are presented in **Figure 1**. Within these structures, multiple interaction sites, with various ligands and receptors (**Figures 1A, B**), lead to a large spectrum of functional facets. Since HMGB1 and the complement proteins in the focus of the present study turn to be multifunctional, the reader will be referred to complementary reviews describing each molecular system in more detail.

## 2 Molecular Bases on Complement and HMGB1 Multiple Functions, with a Complementary Focus on Alarm Raising, Amplification and Dampening

### 2.1 Alarm Signals Raised by Early Steps of Complement Activation and Its Immediate Amplification Loop at the C3 Convertase Level

**Figure 2:**
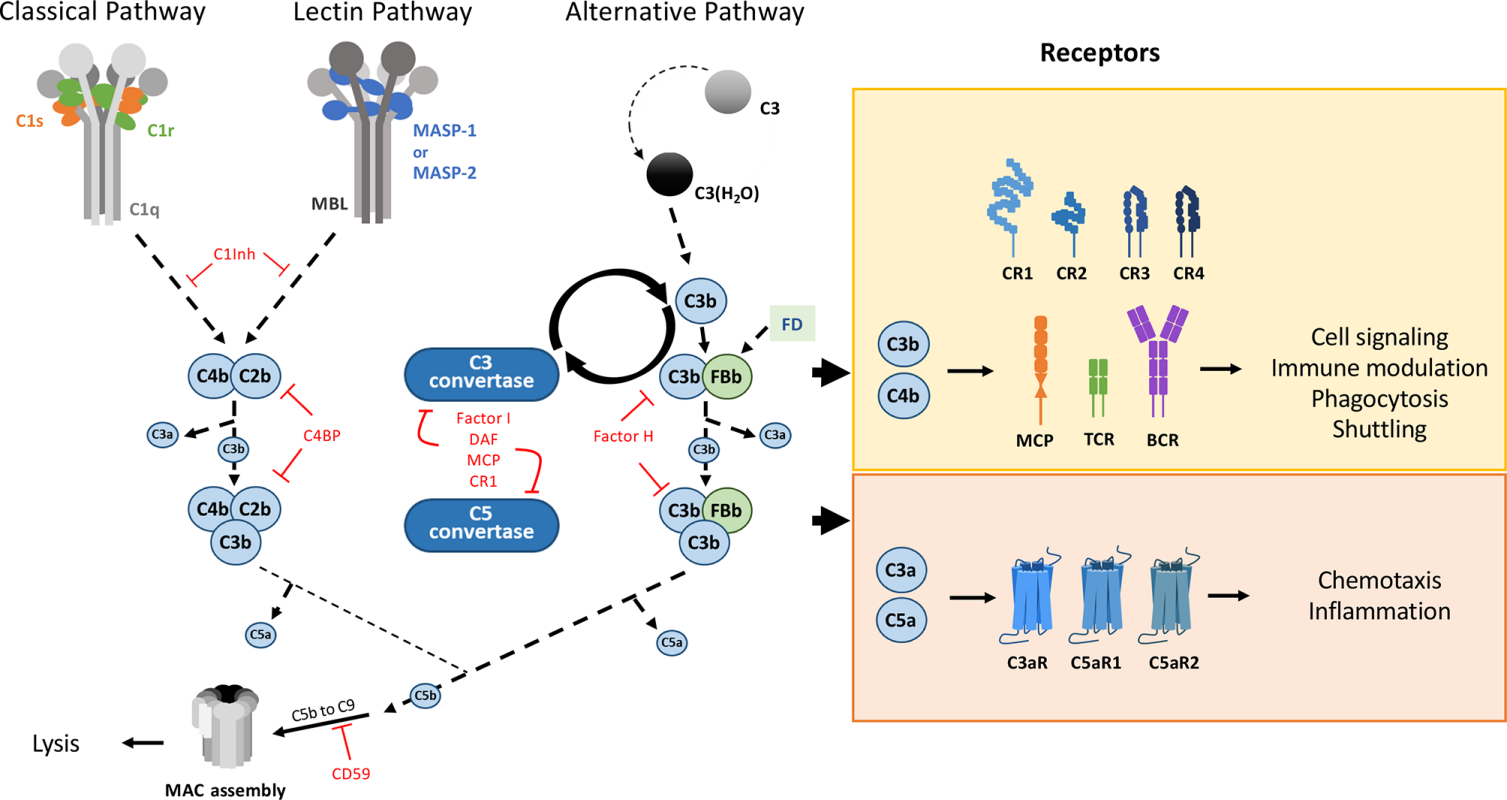
The complement system main pathways and ‘classical’ canonical functions. Triggered by proteolytic cascades, this defense system gets activated and amplified ‘on site’, raising alarm signals through different specific receptors, under the control of numerous regulators. The classical pathway (CP) and lectin pathway (LP) are activated by the C1 complex and the collectin-MASPs complexes, respectively. Collectins (CL) are pattern recognition molecules with collagen-like domains that recognize specific carbohydrate or acetylated surface motifs: MBL, ficolins or collectins recently discovered in the liver (CL-10), kidney (CL-11) or placenta (CL-12) (4). By cleaving C4 and C2, C1s (CP) and MASP-2 (LP) generate a C3 convertase. The (AP) is activated by the spontaneous hydrolysis of C3. All three pathways lead to the formation of the C3 convertase (C4b2b for CP and LP and C3bBb for AP), thus cleaving C3 into C3a and C3b. The amplification loop of the AP C3 convertase leads to massive C3b deposition, covalently bound to the surfaces, targeting them for phagocytosis. The cascade further proceeds to the formation of the C5 convertase, which allows the formation of the Membrane Attack Complex (MAC) on the target surface leading to its lysis. The complement cascade is modulated by various inhibitors (C4BP, C1inh), regulators such as factor H (FH), factor I (FI) that destabilize the C3 convertase (FH, DAF) or cleave C3b (FI assisted by CR1 and MCP (CD46) co-factors). Other regulators limit the final MAC assembly, such as CD59, vitronectin, clusterin … Almost all fragments produced by this enzymatic cascade are bioactive. The C3a and C5a anaphylatoxins mediate chemotaxis and inflammation through their respective receptors, C3aR and C5aR1, C5aR2. Receptors involved in immune modulation depend on the immune cell: CR1, CD46 and TCR on T cells, CR2 and BCR on B cells, recognizing inactivated C3b derivatives. CR1/2/3/4 are also involved in adhesion/shuttling from red blood cells towards the liver and spleen, for example. Only the cleavage products are shown on this simplified scheme, and they follow here the recent recommendation regarding C2 nomenclature (5). Molecule schemes are not adjusted to the same scale.

The main canonical extracellular functions of the complement system are illustrated in a simplified way in **Figure 2**. According to this “classical” view, this system stands as a central component of the early, innate immune response against pathogens, characterized by the initiation of an extracellular proteolytic cascade (6). It comprises more than 50 soluble and surface proteins, the latter including regulators and receptors. Within the activation cascade, which needs to be locally activated and tightly controlled, many of the soluble proteins are produced as inactive precursors, which need a specific enzymatic cleavage to get activated. In terms of biological activities, the complement proteolytic cascade ultimately results in opsonization and degradation of the recognized target as well as signaling towards local and immune cells through surface receptors. There are three complement pathways that differ in their initiation mechanism: the classical, lectin and alternative pathways. Initiation of the classical pathway (CP) is triggered by the interaction of the recognition molecule C1q with the Fc constant domain of immunoglobulins IgM or IgG or with other target patterns on pathogens or altered cells (7, 8). C1q is associated with a duplet of two proteases, C1r and C1s to form the C1 complex (C1qC1r2C1s2). Upon binding of C1 to a target surface, C1r will autoactivate and cleave C1s (7). Activated C1s is then able to trigger the CP proteolytic cascade by cleavage of its canonical substrates C2 and C4. This will lead to C3 cleavage (by the CP C3 convertase) and finally to immune activation, inflammation, opsonization and eventually lysis of the pathogen. Covalent attachment of the large C3b cleavage product to the target surface through a thioester bond ensures the spatiotemporal control of the opsonization, whereas the smaller soluble C3a fragment will mediate more distant chemotactic and inflammatory signaling. C3 is the central element common to all pathways and the starting point of an amplification loop (9) (**Figure 2**). The principle of the amplification is that C3 convertases cleave many C3 molecules, and that the multiple surface C3b produced bind complement factor B (FB), which will then be cleaved by factor D (FD), to generate more C3 convertases. Amplification at this level is very effective since, at a concentration of about 1.2 mg/ml, C3 is among the most abundant plasma proteins in circulation. Therefore, the regulation of the C3 convertase amplification process is essential for the control of complement activation and its dysregulation can have pathological consequences (10). Several receptors mediate complement signaling and complement regulators modulate its trigger (e.g; C1inhibitor) or amplification, by down-regulating of the C3 or C5 convertases (see **Figure 2**). Since the complement system and its activation have already been presented in an article on the present research topic (11), and extensively reviewed previously, the reader may learn more functional and molecular details elsewhere (6, 10–16).

In addition to the “classical” functions which were briefly mentioned above, new functions of the complement proteins are being discovered (17), which are independent of the extracellular activation of the complement cascades. Initially discovered in immune T cells (18, 19), these non-canonical functions are now becoming a wider set of exciting discoveries (20). As will be later cited, some of these new functions include crosstalk with HMGB1, Toll-Like receptors (TLRs), the inflammasome, the coagulation and contact systems, etc…

### 2.2 Alarm Signals Associated With HMGB1 And Their Delayed Paracrine/Autocrine Amplification Mechanisms

HMGB1 is a major intracellular protein, initially discovered as part of a family of DNA-binding proteins remodeling chromatin, involved in the regulation of transcription, replication and repair (21). The spectrum of its functional facets is now largely extended (22). For example in the context of septic shock, its determinant impact has been early observed since HMGB1 injection was fatal whereas its neutralization by antibodies could rescue mice challenged with LPS (23–25). Extracellular HMGB1 is therefore recognized as one of the DAMPs which act as major mediators in immunity (26). Indeed, HMGB1 may be passively released from damaged tissues, late-apoptotic and necrotic cells, as well as actively produced (through a non-canonical secretion pathway) by activated immune cells or even by neurons, as part of inflammatory processes (27–29). In contrast to the immediate activation of the complement system, the secretion of HMGB1 is often delayed, hours after the initial stimulation (23, 30), and its level often remains elevated days after in contexts of chronic inflammation associated with auto-immunity, infection and cancer. HMGB1 persistence maintains and worsens inflammatory disorders, contributing to disease progression through distinct pathways, which depend on its location and diseases context (31). HGMB1 was also the first convincing determinant identified for sterile inflammation, and a key player at the crossroad between innate and adaptive immunity, through the HMGB1 secretion by dendritic cells in response to an initial maturation stimulus, in order to sustain their maturation, and for activation of T lymphocytes (32).

**Figure 3:**
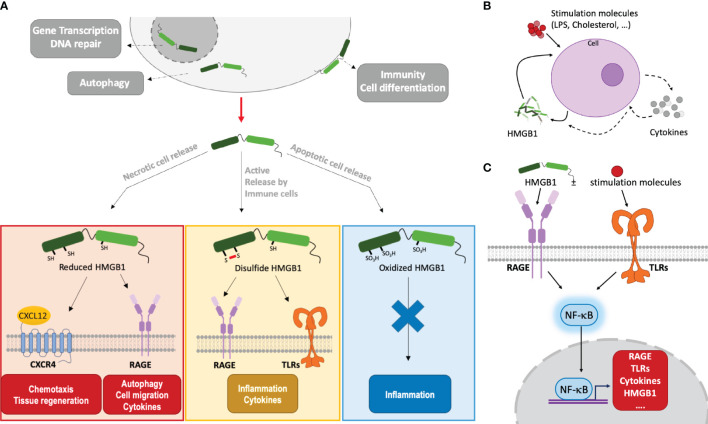
HMGB1 functions depend on its localization/environment, oxidation state and its alarm signaling involves autocrine and paracrine amplification loops. **(A)** HMGB1 effects depend on its localization and oxidation state. Mainly located inside the nucleus, HMGB1 binds DNA and modulates nucleosome stability, thus regulating gene transcription. In the cytoplasm, it plays a role in autophagy. When actively or passively released outside cells, HMGB1 takes part in immunity, inflammation and cell differentiation. HMGB1 can stimulate immune cells (leukocytes, macrophages…). The HMGB1 redox state evolves in relation to its context. Fully reduced, HMGB1 binds to CXCL12 to promote cell recruitment and migration by activation of CXCR4 at the cell membrane. Or, by binding RAGE, it is internalized and it can promote autophagy, cell migration and cytokines synthesis. Through RAGE and TLRs signalization, disulfide HMGB1 induces inflammation and cytokines secretion. When fully oxidized, HMGB1 does not interact anymore with the receptors and may enhance immune tolerance. **(B)** HMGB1 amplifying feedback loop. Under different stimuli, a cell (macrophage, leukocyte, smooth muscle cell, …) can produce and secrete HMGB1 and its receptors directly or *via* the production of cytokines. Once HMGB1 is produced and released, a positive feedback loop is created to enhance its production at the protein level by the initial cell or by neighboring cells, hereby amplifying its effects. This is associated to the secretion of cytokines and other inflammatory molecules. **(C)** NF-κB mediated amplification of HMGB1 and its receptors. By interacting with RAGE and TLR receptors, HMGB1, often in complex with stimulating molecules, will activate the NF-κB pathway. This will induce the transcription of HMGB1, its receptors, and cytokines, leading to an amplification of the inflammatory response.

On a molecular side, the HMGB1 modular structure contains two DNA-binding boxes (A-box and B-box) as well as a C-terminal negatively charged ‘acidic’ tail, strongly enriched in aspartic and glutamic acid residues (**Figure 1A**). HMGB1 action is finely tuned by its associations and localization, which are modulated by post-translational modifications (33, 34). For example, the oxidation state of its cysteines (two in the A-box, one in the B-box) modulates its biological activity in the extracellular environment (**Figures 1**, **3**) (35). Only fully reduced HMGB1 and the non-oxidizable HMGB1 mutant 3S, in which three serine residues replace cysteines, induce chemotaxis by binding CXCL12 through the CXCR4 receptor [maintained at the surface by the CXCL12/HMGB1 heterodimer (36)], and recruit stem cells to orchestrate tissue regeneration (37) (**Figure 3**). Besides this particular activity, the signaling activity of HMGB1 and its complexes (with nucleic acids, LPS, …) is mainly mediated by the receptor for advanced glycation end products (RAGE) and TLRs, although a larger range of receptors may be considered (38, 39). Regarding the proinflammatory function, it is interesting to note that the two HMGB1 boxes have opposing effects when administered separately: the B-box stimulates cytokine secretion whereas the A-box inhibits this effect (30, 39) (**Figure 1A**). Consistently, a recent study observed anti-HMGB1 IgM neutralizing antibodies directed against the B-box in healthy individuals (human and mouse), suggesting a possible feedback loop limiting the level of its pro-inflammatory effect in a healthy state (40). Because HMGB1 can be internalized, and thus reach internal TLRs, it is seen as both a major inside- and outside-cell alarmin (41). Internalization of HMGB1 and HMGB1-partner molecule complexes depends on RAGE and, notably, is inhibited by the recombinant HMGB1 A-box protein (27).

Autocrine or paracrine positive feedback loops involving potent HMGB1-mediated signal amplifications are observed in a variety of contexts (42) (**Figure 3B**). For example, TNFα is secreted by endothelial cells or macrophages stimulated by HMGB1, and, in turn, TNFα will induce HMGB1 secretion from these cells. Similarly, smooth muscle cells, which are normally not HMGB1 producers, can start to secrete HMGB1 when challenged with cholesterol. In turn, once activated by HMGB1, the smooth muscle cells proliferate, migrate and … secrete more HMGB1! (43). Positive feedback loops may involve NFκB activation by HMGB1 through RAGE and/or TLRs. In turn, NFκB will increase the surface expression of these HMGB1 receptors and the production of cytokines, including HMGB1 (42) (**Figure 3C**). Local amplification can also proceed through infiltrated macrophages and leucocytes which, upon HMGB1-mediated activation, will secrete more HMGB1 (and TNFα) (42). In a context of sterile inflammation through acetaminophen challenge, the amplification may also be driven by neutrophils, which are massively attracted to the necrotic site in an HMGB1- and RAGE-dependent manner and exacerbate tissue injury (44).

### 2.3 Possible Cross-Amplification Through Enhancement of HMGB1 Secretion by C3a/C5a Anaphylatoxins, HMGB1-Mediated CP Activation and More Indirect Processes

**Figure 4:**
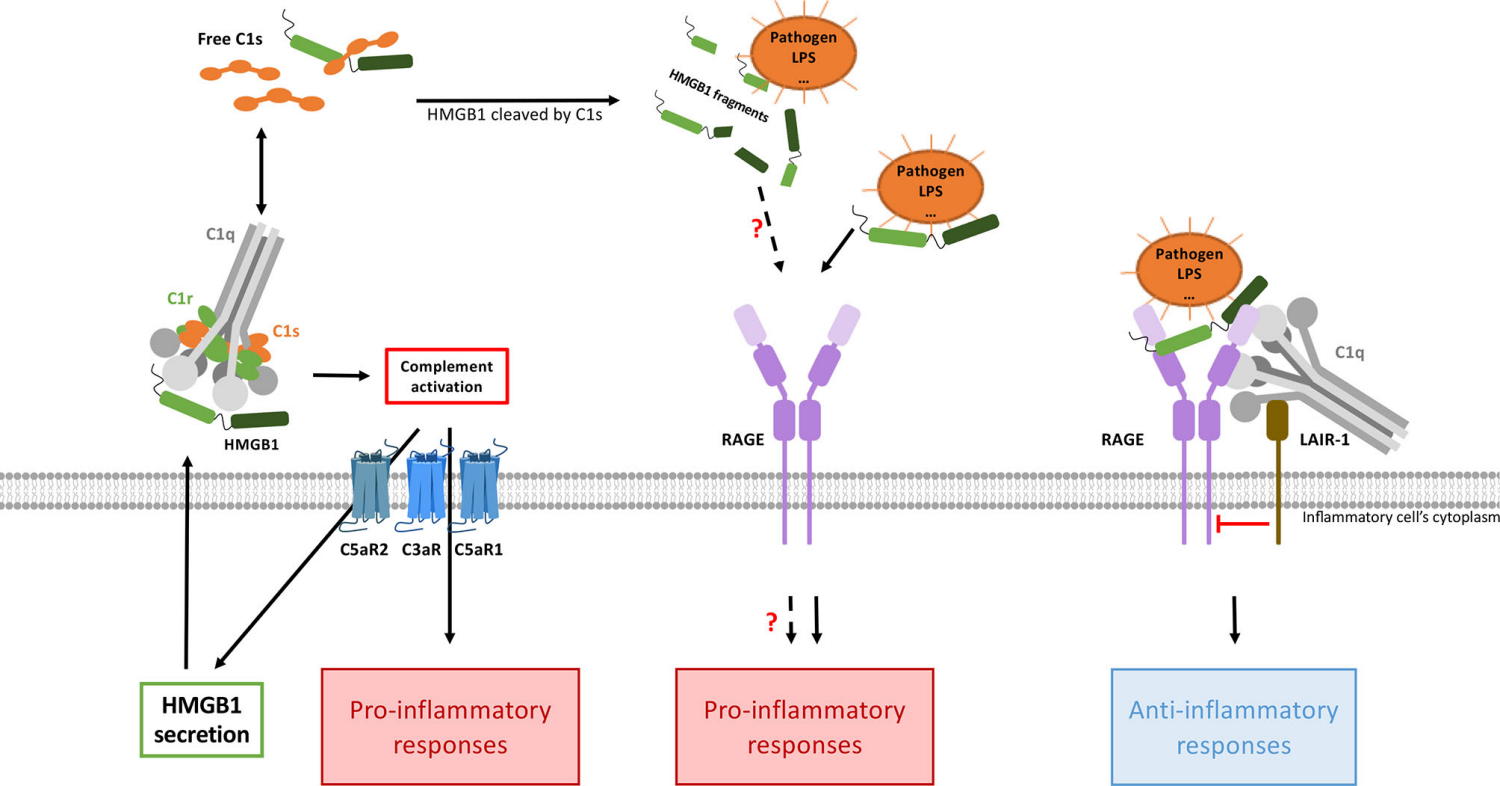
HMGB1 and complement interplays combine pro- and anti-inflammatory responses. CP activation (possibly directly mediated by HMGB1) will produce anaphylatoxins, which enhance HMGB1 secretion (left). In this context, the pro-inflammatory responses triggered by the two pathways are additive and cross-amplified. On the other hand (right), when C1q bridges LAIR-1 and HMGB1-RAGE, then LAIR-1 recruits the SHP-1 phosphatase which will shut down the RAGE-mediated inflammatory signaling, thereby dampening inflammation. Activated C1s can also cleave HMGB1, but the functional impact of the HMGB1 cleavage fragments need to be investigated. By inhibiting HMGB1internalization through RAGE (not shown here), C1q can also limit the extent of intracellular TLR activation.

As seen above, several feedback loops tend to auto-amplify the alarm signals generated at the levels of HMGB1 and C3a. Whether further cross-amplification may occur between the two systems is, therefore, the next question to address (**Figure 4**). Although this certainly depends on the specific context, it is interesting to note that several independent publications mention C3a or C5a mediated enhancement of HMGB1 secretion. Intriguingly, C3a was shown to tightly bind the RAGE receptor (45). Since C3a and the HMGB1 A-box are small helical domains (**Figure 1C**), and share this RAGE binding property, it was tempting to look for some 3D similarity between the two, but in fact the overall similarity is very limited (**Figure 1C**). Furthermore, the reported functional impact of this C3a-RAGE interaction was to increase IFN-α production by human peripheral blood mononuclear cells in response to unmethylated cytosine-guanine-rich DNA (45), but this remains quite poorly explored (10).

A so-called “C3/HMGB1/TGF-β1 pathway” was reported in two publications investigating the pathogenesis of diabetic nephropathy and its associated chronic renal fibrosis (46, 47). In response to C3a stimulation (provided by macrophage infiltration into renal interstitial tissues), HMGB1 translocates from nucleus to cytoplasm in primary renal tubular epithelial cells, and expression of HMGB1 and its receptor TLR4 gradually increases in their cytoplasm from 24 to 48 h after C3a stimulation. This will trigger epithelial to mesenchymal transition through the HMGB1/TLR4/p65/TGF-β1 signaling pathway (46). These effects have been inhibited by the grapefruit component GSPE (46) or the specific MiR-92d-3p miRNA in a renal tubular epithelial cell line (HK2) (47).

Although the role of the C5aR2 receptor in inflammation and disease remains a less studied and controversial topic, several independent publications describe that C5aR2 is critically involved in LPS-induced HMGB1 secretion (48). Indeed, macrophages that only express C5aR2, without C5aR1, show a marked HMGB1 production upon C5a stimulation, associated with signaling through MEK1/2, JNK1/2, and PI3K/Akt activation pathways. The critical role of C5a/C5aR2 interaction was later deciphered as activating the NLRP3 inflammasome and then HMGB1 release by macrophages by raising the expression of PKR *via* MEK/ERK and interferon (IFN) pathways (49). Reversely, an anti-HMGB1 antibody (Ab) reduced the C5a/C5aR2 interaction–mediated caspase-1 activation and IL-1β secretion by macrophages (50). C5a also triggers the release of HMGB1 by primary human neutrophils and plays, together with HMGB1, significant roles in the context of antineutrophil cytoplasmic Ab-induced neutrophil activation (51).

In turn, HMGB1 has been shown to activate the classical complement pathway in an Ab-independent manner, by directly binding to C1q. This process may drive sterile inflammation (52) and contributes to cross-activation in the complement C1/HMGB1 interplay (**Figure 4**).

Several lines of evidence suggest that cross-amplification may also be indirectly mediated by cytokines, such as IFNγ or TNFα. For example, Ab-mediated complement activation or HMGB1 can contribute to enhance IFNγ secretion (53, 54), which in turn can enhance C1s secretion (55, 56) or NLRP3 inflammasome signaling (53). TNFα, which is amplified by HMGB1 in response to LPS (57), may trigger C3 or C1s secretion (58, 59). Of note, it was early shown that IFNγ and TNFα are both secreted by human T cells as a response to C1q-bearing immune complexes, but not by non-opsonized complexes (60). This is also true for C3a and C3b, which are decisive drivers for a Th1 response in CD4+ T cells, with the induced secretion of IFNγ and TNFα (61). It was also shown that a crosstalk between TLRs and anaphylatoxins or C3b-coated antigens shape antigen-presenting cells towards inducing such a Th1 effector response (61).

### 2.4 Dampening Inflammation Through a Regulatory Anti-Inflammatory Feedback Loop Involving Complement C1, LAIR-1 And HMGB1

Inhibitory pathways are needed to dampen inflammation and limit inadequate autoreactivity. Interestingly, recent discoveries have opened a historical entry point for a non-inflammatory crosstalk between C1q and HMGB1 (62, 63). Such studies contribute to better understand the complex disease processes in systemic lupus erythematosus (SLE), the first disease context that will be taken as an example in the next section.

These regulatory roles of C1q are mostly mediated through its interaction with Leukocyte-Associated Immunoglobulin-like Receptor-1 (LAIR-1). LAIR-1 is an inhibitory receptor expressed on a wide range of human immune cells such as NK cells, T cells, B cells, monocytes, neutrophils, basophils and mast cells (64–71). Its cytoplasmic region contains two amino acid sequences corresponding to immune receptor tyrosine-based inhibitory motifs (ITIMs), which mediate its immune inhibitory activity (64). C1q can interact with LAIR-1 through its collagen-like region (CLR) (72) (**Figure 1B**). As a first major interplay, the combined action of C1q and HMGB1 was shown to regulate human macrophages polarization (62): in lipid rafts at the monocyte surface, the multivalent C1q molecule can bridge RAGE/HMGB1 on one side, and LAIR-1 on the other side, which triggers the phosphorylation of the two ITIMs in LAIR-1, and the consequent recruitment of the SHP-1 phosphatase (**Figure 4**). Although HMGB1 alone polarizes macrophages to the M1-like inflammatory type, through RAGE phosphorylation, the recruitment of SHP-1 in the cross-talk with C1q and LAIR-1 will dephosphorylate the proximal RAGE receptor, and macrophages get polarized into a M2-like type. If C1q is too low, because of rare genetic deficiencies or immune-complexes-driven consumption, and if HMGB1 level is high, then this inhibitory control of the macrophage is ineffective, leading to M1 polarization, impaired clearance of apoptotic cells, and exposition of the adaptive immune system to numerous autoantigens, therefore generating auto-Abs. Further studies from the group of Betty Diamond have shown that HMGB1 plus C1q increase the secretion of pro-resolving lipid mediators by activated monocytes (63). On the same line, C1q/LAIR-1 interaction was shown to inhibit TLR activating signals to maintain monocyte tolerance (73), to inhibit monocyte to dendritic cell differentiation and to suppress INF-α production by plasmacytoid dendritic cells (pDC) (74).

On another line, C1q was shown to inhibit RAGE-mediated internalization of HMGB1, which may also directly contribute to its regulatory role (62). C1s was also shown to cleave HMGB1, which may reduce its immunogenicity. This proteolytic cleavage also reduces the HMGB1 property to amplify LPS-mediated proinflammatory cytokine secretion from monocytes, macrophages and dendritic cells (57, 75). All these properties may contribute to the non-inflammatory role of the C1 subunits in its cross-talk with HMGB1, but the underlying molecular mechanisms remain partly unexplored.

Altogether, these observations suggest that the interplay between HMGB1 and C1q/C1s can raise and amplify initial alarm signals, and then dampen the raised inflammation.

## 3 Examples of HMGB1 and Complement Dysfunctions and Crosstalk in Inflammatory Diseases

### 3.1 Exacerbating Instead of Dampening Inflammation in SLE Through HMGB1 and Complement Crosstalk

SLE is an emblematic chronic multifactorial auto-immune disease where the two alarm systems likely crosstalk and contribute to immune dysregulation (62). This disease is characterized by the production of multiple autoantibodies (auto-Abs). Regarding its etiology, it combines genetic predisposition and environmental factors that alter immune regulatory processes. It presents alternative periods of illness (flares) and wellness, and may affect different tissues. The clinical symptoms thus range from skin rashes, chronic fatigue, and arthritis to the more severe nephritic and neurological involvements (12). Disease evolution into lupus nephritis (LN) is the most common cause of morbidity and mortality, by progressing to end-stage renal failure (76). LN remains scientifically challenging to understand and predict, since it involves multiple pathogenic pathways including altered cell death, auto-immune complexes deposition, complement activation, and inflammatory flares (77).

#### 3.1.1 SLE, Autoantibodies and Complement

Defining the role of the CP proteins in SLE is complex and has been recognized as a classical paradox in the field (78) since the system dysfunctions when these proteins are deficient (genetically or by consumption) or over-activated (12).

Since the discovery that deficiencies in CP proteins are the strongest genetic risk factors for developing SLE (79, 80), a link has been established between CP protein abnormalities and this complex disease. Cases of complete C1q, C1s, or C1r deficiencies (79–81), or of mutated C1q, which can no longer associate with its cognate C1r and C1s proteases (82), are all associated with human SLE with mortality or morbidity at a young age. This was initially explained by the impaired role of the CP components in the clearance of immune complexes and “self” debris such as apoptotic cells, known as the waste disposal hypothesis (83). More than 100 cases of complete C1q deficiency are described now, with variable clinical presentations, often sharing SLE or SLE-like disease and recurrent bacterial infections (84). Fewer (dozen) cases are described with complete C1s deficiency, maybe because recurrent infections and mortality at a young age are common in these patients. Altogether, these clinical data suggest that the CP proteins are protective from developing SLE.

**Figure 5:**
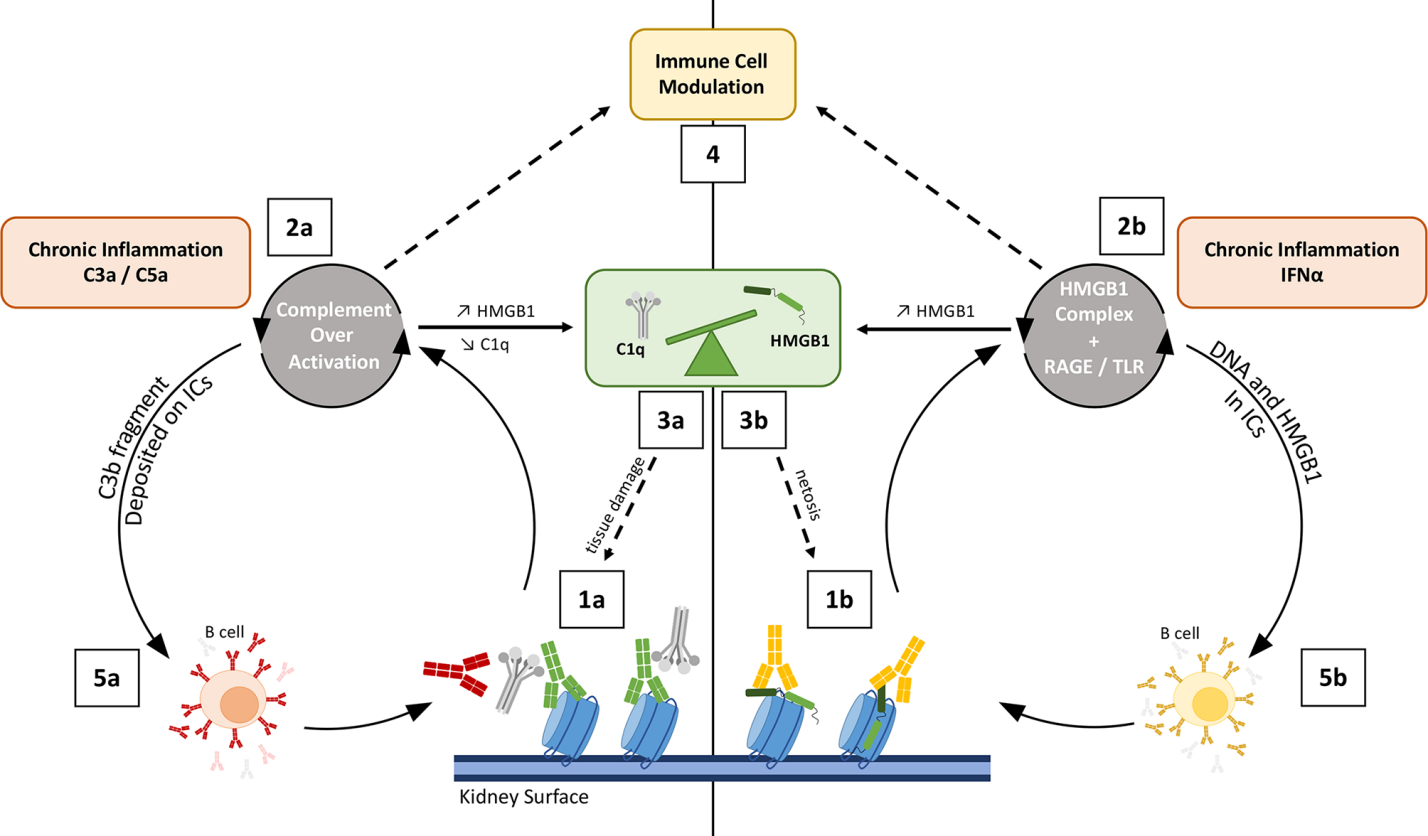
Schematic view on how complement and HMGB1 may co-amplify tissue damaging feedback loops in SLE/LN. Left: Complement overactivation (1a to 5a); Right: HMGB1-mediated impact (1b to 5b). On the left side, complement over-activation (through C1r/s, not shown) by intrarenal deposited immune complexes is enhanced by anti-C1q (red) (1a). This leads to C3a-C5a driven inflammation (2a) and renal tissue damage, with impaired clearance because of C1q and C3 depletion, therefore releasing more nucleosomes (3). On the right side, HMGB1 forms complexes with nucleosomes (1b), LPS or cytokines. These HMGB1 complexes enhance inflammation in the kidney or in the skin of lupus patients, and activate IFN-α production (a driving cytokine in lupus pathogenesis, 2b). Both HMGB1 and C3a/C5a can stimulate HMGB1 release by activated cells (2a, 2b); HMGB1 is also passively released by complement-damaged cells (3a), and through increased netosis induced by HMGB1 (3b). The consequent imbalance effect on C1q and HMGB1 hampers proper inflammation resolution (see **Figure 6**) (4). On both sides, the process stimulates B-cells to produce more auto-Abs, which fuels the two combined vicious circles at the first steps (5a, 5b). Different types of auto-Abs are illustrated, with possible functional interference: anti-nucleosome (green), anti-C1q (red), anti-HMGB1 (orange).

However, since SLE is the disease with the largest number of identified auto-Abs, auto-immune complexes deposited on the renal surface can over-activate the complement CP, inducing strong tissue damage and profound C1q, C3 and C4 consumption (**Figure 5**). Moreover, anti-C1q auto-Abs, which are found in more than 40% of the LN patients (85), can alter C1q functions and contribute to the intrarenal complement over-activation and tissue damage (86, 87).

Although anti-C1q auto-Abs are often associated with LN, anti-C3b auto-Abs were found more specific, but less sensitive, suggesting the possible use of a combination of both auto-Abs as biomarkers to follow LN activity in SLE patients (77). More recently, C3 and C4 activation products, present in plasma and/or at the surface of blood cells, have been proposed as diagnostic markers with a tight correlation with SLE disease activity (88).

Although apparently contradictory, these different observations reveal that the function of the CP proteins is essential but needs to be tightly balanced and controlled. Exploring this paradox has led to the discovery of many other functional facets of the C1q molecule (89), as reviewed recently in (90–92). The role of the C1 proteases is also to be deciphered outside of their classical role, where complement-C3d deposition on immune complexes (ICs) results in enhanced auto-Abs production by B cells (93, 94) (**Figure 5**).

#### 3.1.2 SLE, Autoantibodies and HMGB1

Pathological roles in SLE for the alarmin HMGB1 and anti-HMGB1 auto-Abs were proposed recently (95). For more details, the reader is referred to a recent mini-review and references therein (96).

In SLE patients, higher HMGB1 serum concentrations are reported, increasing during active disease and remaining elevated even during anti-inflammatory treatments (97). A significant, positive correlation was found between HMGB1 mRNA and SLE disease activity index (SLEDAI) (98). Besides its global contribution to the pathogenesis of SLE, HMGB1 was reported to play a particular role in LN, possibly serving as a marker of disease activity in patients with renal impairment (95). In keeping with this question, it has been proposed recently that serum HMGB1 (in a context of pediatric SLE) or microparticles containing HMGB1 observed in the urine of patients may be useful as biomarkers of LN (99, 100). Interestingly, another study focusing on auto-Abs directed against HMGB1 A-box concluded about their potential interest as a biomarker for SLE, especially for the prediction of disease activity, and not specifically related to the kidney (101).

As for C1q, it is proposed that HMGB1 impacts both the innate and adaptive immune cells in SLE (96). HMGB1 may impair apoptotic cells clearance, strongly stimulating the secretion of proinflammatory cytokines like IFN-I by pDCs. It can impact cell death processes (e.g. increase neutrophil netosis).

We will mainly refer here to further observations not reported in the reviews cited above. In the particular context of LN, TLR2 has been shown to regulate glomerular mesangial matrix deposition through the activation of the MyD88/NF-κB pathway though interactions with HMGB1 (102). HMGB1 upregulation in SLE correlates with the activation of dendritic cells, targeting myeloid dendritic cells *via* the upregulation of the mTOR pathway, for example. This signaling pathway leads to the exposure of HLA-DR, CD40, and CD86 on dendritic cells and enhances the secretion of IL-1β, IL-6, and TNF-α cytokines (103). Intriguingly, high molecular weight covalent complexes including HMGB1 circulate in the blood of SLE patients at a significantly higher concentration than in healthy patients (104).

HMGB1 also takes part in the stimulation of BAFF (B-Cell-Activating Factor) and thus auto-Ab production (105, 106), introducing a vicious circle as illustrated in **Figure 5**. Indeed, ICs containing DNA and HMGB1 can promote TNF-α and BAFF production through RAGE, leading to B cell hyperactivity (105). HMGB1 gene and protein expression is significantly increased in SLE CD4(+) T cells compared to controls, and it could impact the DNA methylation level (98).

#### 3.1.3 HMGB1 and C1q or C1s Crosstalk in SLE

**Figure 6:**
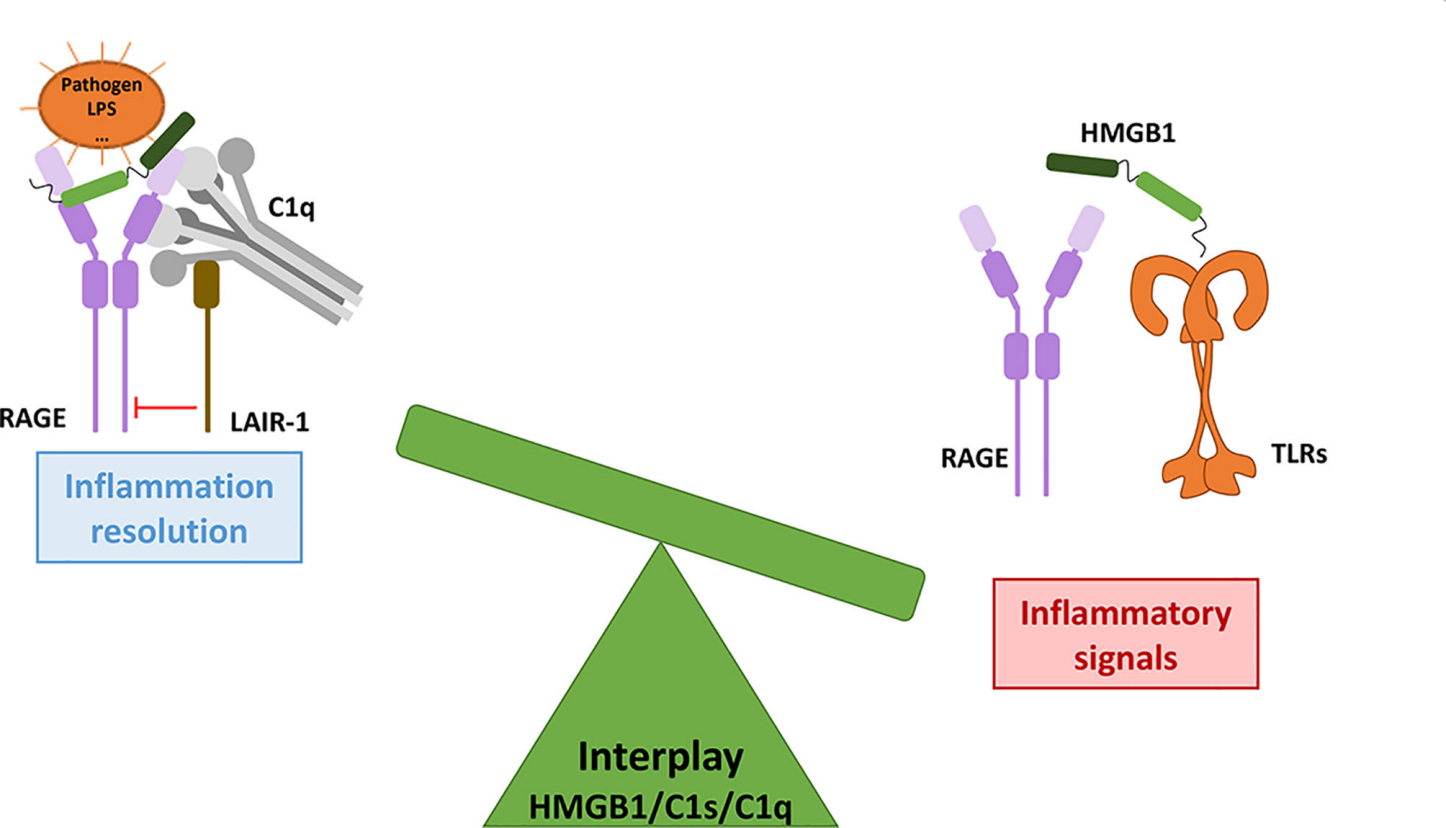
The HMGB1/C1s/C1q interplay modulates the inflammatory signals from initiation to resolution. As a molecular switch, C1q can dampen the inflammatory signals triggered by RAGE/HMGB1, through LAIR-1 inhibitory signaling. The role of C1s in this interplay remains to be further investigated.

Numerous studies have contributed to elucidate the role of C1q in SLE. Among them, two reported a C1q interplay with HMGB1 towards inflammation resolution and immune balance, involving the LAIR-1 and RAGE receptors (62, 63), as described previously (**Figure 4**). In addition, the cleavage of HMGB1 by C1s was proposed to suppress surface autoantigen epitopes (57). The simplified integrated overview presented in **Figure 5** illustrates the interconnections between the functional effects of CP proteins and HMGB1, with possible cross-amplification of inflammatory vicious circles in a LN disease context. For example, in correlation with disease severity, elevated circulating HMGB1 seen in SLE patients can bind to nucleosomes released from damaged cells, and be part of ICs (because of anti-HMGB1 and anti-DNA auto-Abs) (107). These ICs will trigger complement activation, which consumes C1q and releases anaphylatoxins, which upregulates HMGB1. This process induces a molecular imbalance between HMGB1 and C1q (**Figure 5**). This imbalance (**Figure 6**), together with the downregulation of LAIR-1 on pDC in pediatric SLE (108) or at the surface of B cells (109), will limit the feedback control through these molecules shown in **Figure 4**. C3 fragments deposited on the surface of ICs and HMGB1 present in nucleosomes complexes will stimulate B cells to produce more auto-Abs.

Hyperactive T cell responses occur in SLE patients, with different co-signals. Indeed, ICs and late complement activation components trigger the activation of Syk tyrosine kinase co-signal in CD4+ T-cells through FcγRIIIa, which up-regulates TLR and HMGB1 expression, as well as the expression of IFN pathway genes (110). In addition, the deficit of spatial memory in neuro-SLE patients is a consequence of dendrite destruction by microglia. HMGB1, decorating synapses on neurons damaged by anti-neuron antibodies, enhances C1q binding which activates the microglia and targets the dendrite for destruction and remodeling (111). Both the central and peripheral nervous systems can be affected, leading to cognitive impairments.

### 3.2 C1, Complement or HMGB1 Dysfunction and Possible Crosstalk in Severe Periodontitis-Like Diseases, With Tissue Degradation Associated With Chronic Inflammation and Dysbiosis

We now come to a context of inflammatory disorders which affect the periodontium, which is the tissue supporting the teeth, which includes different structures such as gingiva, cementum, periodontal ligament and alveolar bone. Periodontitis is the prototype of disease where destructive inflammation and microbial dysbiosis are reciprocally reinforced in a complex interplay. In severe cases, when inflammation persists unresolved, the exacerbated host reaction can lead to the degradation of the periodontium, namely periodontitis. Connective tissue damage and loss of alveolar bone are mediated by a dysregulated and excessive inflammatory response, which involves components of both innate and adaptive immunity, including the complement system (112, 113). Persistent inflammation leads to microbial dysbiosis in favor of bacteria surviving in these conditions and using tissue breakdown products as nutrients. Within a vicious circle, the imbalanced composition of oral bacteria will trigger more inflammation, leading to tissue permeability. Therefore, oral bacteria may disseminate in the whole body, which will extend the location of microbial dysbiosis disorders. Importantly, this chronic inflammatory context is associated with an elevated risk of systemic conditions, which reinforces the need to better understand and treat periodontitis-like diseases (114).

#### 3.2.1 Complement in Ehlers-Danlos Syndrome Periodontal Subtype and in Periodontitis

Our interest in this disease context opened through a collaboration with scientists from Innsbruck Medical University, who discovered heterozygous mutations in C1 subunit genes *C1R* and *C1S*, which were clearly associated with a specific periodontal subtype of Ehlers-Danlos Syndromes (EDS) (115). EDS is an umbrella term for a group of inherited connective tissue disorders, characterized by joint hypermobility, skin hyperextensibility, and tissue fragility. A predominant clinical feature of periodontal EDS (pEDS) is early severe periodontitis and tooth loss or resorption. During childhood, before the onset of periodontitis, a common manifestation for this very rare disorder is the lack of attached gingiva, consistently observed only in children who inherited the familial pathogenic pEDS variant (116). Early bruising was another symptom commonly associated with children inheriting the pathogenic pEDS variant.

Identifying mutations in the *C1R* or *C1S* genes is among the criteria needed to diagnose pEDS (117). What could be the link between the heterozygous mutations in the *C1S* and *C1R* genes and this very rare syndrome? Interestingly, periodontitis can occur in this particular pEDS context without strong pathogenic colonization in gingival pockets. This observation therefore suggests that alterations in C1r or C1s functions might trigger sterile inflammation in this specific pEDS context. Two studies aimed to explore the molecular consequences of these specific mutations in C1r and C1s (118, 119). Defective secretion of these proteases is always observed in the presence of all pEDS mutations, with the only exception of a particular mutation in C1r which affects a C1q-binding residue (118). A common feature is that the altered C1r or C1s proteases, if secreted, will not assemble into the extracellular C1 complex. Therefore, this suggests that their activation and activity will escape from the physiological control associated with the C1 context. In cases of the mutations affecting the C1r coding sequence in pEDS patients, the C1s protease is intact but its level of activation is significantly increased in the supernatant of patient fibroblasts as compared to controls (without activation), which suggests that C1s activity may be involved in the pathological process (118). Further *in vitro* studies, analyzing the impact of the mutations affecting the C1s coding sequence in pEDS patients, unexpectedly revealed that only a C-terminal fragment was secreted during recombinant C1s variant expression in HEK cells. Importantly, this C1s C-terminal fragment was lacking the capacity to cleave C4, which led us to check if this fragment retained the capacity to cleave the non-canonical HMGB1 target, which was the case, although the generated fragments may slightly differ in proportion (119). As a first conclusion to these studies on pEDS mutations, the common observation is that the C1s protease gets constitutively activated, without the need of the canonical C1 trigger, and we suggest that C1s constitutive activity may thus be at least partly responsible for the observed dominant inheritance.

Consistently, we will recapitulate below the current observations on the involvement of complement (and HMGB1 in the next section) in severe periodontitis. Complement proteins are present and produced in the oral cavity. Several clinical observations and pre-clinical intervention studies that collectively suggest that complement is hyperactivated in periodontitis have been recently reviewed (112). In healthy conditions, complement split products are either absent or present in low concentrations in the gingival crevicular fluid. In contrast, complement C1q, FB, Bb, C3, C3a, C3b, C3c, C3d, C4, C5, C5a, C5b and C9 have all been detected in diseased periodontal tissue and in the gingival crevicular fluid from patients with established periodontitis (120). Increased local activation of complement in the periodontal tissues enhances the local inflammatory response, as well as loss of tooth attachment and ultimately bone resorption among the associated clinical manifestations (120). The formation of osteoclasts, the cells responsible for the removal of mineralized tissue, is modulated by C3a and C5a anaphylatoxins, in synergy with IL-1ß (121). Recent studies suggested that the complement system and mast cells are associated with the destructive periodontitis processes of alveolar bone and tooth resorption in cats (121).

In the childhood context, characterized by decidual teeth, we can note that decidual ligaments have been observed to express significantly more complement C1s than permanent ligaments, as well as more laminins extracellular matrix components, with morphological differences between the two types of periodontal ligament tissues (122). Coming back to the pEDS disease context, there may therefore be a possible link between the above observations and the absence of attached gingiva observed in the children who inherited from the pathogenic variant in pEDS families (116).

#### 3.2.2 HMGB1-RAGE Axis and Interplay Between the TLR/Complement Crosstalk and Pathogens in Periodontitis

As recently reviewed (123), it has been proposed that HMGB1 plays a role in the exacerbation and propagation of the oral inflammatory disorders. Infection promotes HMGB1 secretion from periodontal tissue, and the secreted HMGB1 is involved in the lingering or aggravation of periodontitis. Anti-HMGB1 antibodies were shown to attenuate periodontal inflammation and bone resorption in a murine periodontitis model (124). Several oral cells secrete HMGB1 in response to bacterial infections (for example *P. gingivalis*) or stimuli like LPS, butyric acid (which is a metabolite of periodontal pathogens), IL-1β, TNF-α. These include gingival epithelial cells, gingival fibroblasts, human periodontal ligament fibroblasts and of course macrophages. Elevated concentrations of HMGB1 in the gingival crevicular fluid were observed in periodontitis patients, with significant positive correlations between these HMGB1 levels and all periodontal parameters, including plaque index, bleeding index, probing depth, and clinical attachment level. Thus, infection and inflammation promote HMGB1 secretion from periodontal tissue, which further promotes pro-inflammatory cytokine production and osteoclast generation, aggravating periodontitis. RAGE is also highly upregulated in patients with combined type 2 diabetes and periodontitis, as compared to patients with chronic periodontitis. The two occurences of the word patients were not appropriately positioned (125).

Subversion of the complement/TLR crosstalk by pathogens was also described in the context of periodontitis (126). For example, *P. gingivalis* is a strict anaerobic bacterium that requires peptides and hemin for its growth, and thus depends on the continuous flow of inflammatory serum exudates to obtain essential nutrients for survival in the periodontal niche. Its proteases, gingipains, directly cleave C5 to generate C5a, but also degrade C3 and C5b, which inhibits membrane attack complex (MAC) formation. By another subversive mechanism involving TLR2 and C5aR1, the bacteria suppress the macrophage and neutrophil immune functions (126). This C5aR1–TLR2 crosstalk upregulates the production of proinflammatory cytokines (IL-1β, IL-6, and TNF-α), which appear to mediate inflammatory bone loss in a murine model of experimental periodontitis, as well as dysbiosis. Finally, deepening of the periodontal pockets provides nutrients and more room for bacterial growth. What about HMGB1 in this TLR/complement crosstalk? In the signaling cascade proposed by (123), HMGB1 is secreted by gingival epithelial cells in response to LPS by TLR2/4 and in response to butyric acid by other receptors. In a paracrine/autocrine mode, extracellular HMGB1 then induces signaling through TLR2, MD2/TLR4 and RAGE in the gingival epithelium, macrophages and periodontal ligaments. As seen before, the complement anaphylatoxins might up-regulate HMGB1 secretion through C5aR2. However, this interplay remains ill-defined for the moment and no figure will be proposed here. Coming back to the context of the C1s mutations studied in the pEDS disease, if the inherited pEDS mutation induces pathological effect because of uncontrolled C1s activity, we suggest that a dysfunction in the C1/HMGB1 crosstalk might be involved in this context, although the details of possible molecular mechanisms remain to be solved (119).

## 4 Concluding Remarks

Complement and HMGB1 are major actors of our defense systems, involving various amplification mechanisms as well as a wide range of surface receptors. Together, they first provide effective danger signals and front lines against external as well as internal insults, and can then switch towards inflammation resolution and repair. Reflecting some contradictory issues in the field, we aimed here to illustrate how their interplay may reciprocally be reinforced for the bad, in disease states, or also for the best, when repair is effective. As suggested in **Figures 5**, **6**, the molecular switch towards inflammation resolution, which involves LAIR1, depends on a fine molecular balance between C1 components and HMGB1, likely a key element in the SLE context.

Complement proteins are mainly and traditionally seen as extracellular factors, but a huge interest has been recently raised in the discovery of intracellular new functions. Conversely, HMGB1 is traditionally seen as an intracellular protein (nuclear, cytoplasmic, …) but various extracellular functions have now been deciphered in the last decades. C1q, HMGB1, C3 (and its activation products) are all therefore multitasking effectors (10, 90, 127). Their different tasks critically depend on the location, post-translational modifications (including proteolytic cleavages) and interacting partners, which the larger C1q molecule can locally bridge. Since these multiple tasks are assigned to different compartments or different steps in the processes, we choose here to avoid the ‘dual role’ or ‘double edged-sword’ mention often used in the case of C1q and HMGB1, although it is clear that in certain pathological dysregulated contexts they may be involved in strong pathological amplification of inflammation and tissue injury.

The reader may find complementary details and further inspiration in previous reviews close to this subject, from the different angles of the complement and TLR crosstalk (126, 128), complement-inflammasome crosstalk (129, 130), complement and cell death (75) or complement and SLE (92). Part of this subject is also related to reviews including non-canonical functions of complement (14, 90) or HMGB1/alarmins (31, 131) or C3 multiple functions (10).

Beyond C5aR2-stimulated HMGB1 secretion (132), publications referring to both HMGB1 and complement remain rare but their number increased recently. They are focused on a specific aspect of the crosstalk, or associated with very severe conditions, as for example Gulf War Illness (133) or hemorrhagic shock (134), or may also relate to the field of neuroinflammation (135). This latter aspect of neurological disorders has not been fully addressed in this review, although alteration of the brain white matter has been observed for at least two pEDS families, which raises the question of the possible impact on the central nervous system (136). On a larger scale, cognitive impairment occurs in 40–90% of SLE patients (111, 137). To better understand the latter condition, the group of Betty Diamond has further explored in a mouse model how C1q and HMGB1 target together neuronal dendrites for destruction, which translates into deficits in spatial memory (137).

Hopefully, this review will shed light on new perspectives on molecular dominos and possible vicious circles combining the two systems. The disease examples cited in this review are likely ‘the tip of the iceberg’. SLE is clearly the more established example, thanks to numerous studies trying to elucidate the paradoxical observation that C1q deficiency is such a high-risk factor to develop this disease. The interplay between complement and HMGB1 could likely play a key role in several other severe disease contexts, in particular inflammatory diseases where the immune pathology is host-driven, including cancer, sepsis, senescence. Even in the current pandemic COVID-19, increased circulating HMGB1 levels were observed (138) and crosstalk between the complement, contact, and coagulation systems contributed to severe pathological consequences of the infection (11).

Many challenges remain ahead. These soluble mediators may be easily ‘invisible’ in studies focused on immune cells. How to decipher the occurrence and impact of various post-translational modifications of HMGB1 in physiological but also pathological contexts? These modifications are not limited to the oxidation state of the cysteines (34). Experimental tricks are needed to deconvolute the impact of extracellular and intracellular contributions, as well as their local or systemic origin. Deciphering new non-canonical functions remains challenging although impressive recent progresses were made on this side. Lastly, a better understanding of these interrelated systems will open the door to new personalized therapeutic strategies including adjunctive treatment, for example to downregulate HMGB1 (139), while new drug developments are performed to control complement and HMGB1 pathological effects, as discussed in recent reviews or research papers (9, 10, 27, 33, 63, 112, 140).

## Author Contributions

CG, ML, NT, and CD-P reviewed the literature or wrote sections of the review article. ML created figures, supervised by CG and VR. CG organized and wrote the main part of the text. All authors contributed to the article and approved the submitted version.

## Funding

Interest in the topic reviewed here and related research work are/were supported by three grants from the French National Research Agency (ANR): DYSALARM (ANR-21-CE14-0066), C1rsinEDS (ANR-16-CE91-0004) and C1qEffero (ANR-16-CE11-0019). ML is funded by a fellowship from the University Grenoble Alpes graduate school (Ecoles Universitaires de Recherche) CBH-EUR-GS (ANR-17-EURE-0003).

## Conflict of Interest

The authors declare that the research was conducted in the absence of any commercial or financial relationships that could be construed as a potential conflict of interest.

## Publisher’s Note

All claims expressed in this article are solely those of the authors and do not necessarily represent those of their affiliated organizations, or those of the publisher, the editors and the reviewers. Any product that may be evaluated in this article, or claim that may be made by its manufacturer, is not guaranteed or endorsed by the publisher.

## Abbreviations

Abs, antibodies; ACAMPs, apoptotic cell associated molecular patterns; Akt, protein kinase B; AMPs, associated molecular patterns; AP, complement alternative pathway; auto-Abs, autoantibodies; BAFF, B-cell activating factor; BCR, B-cell receptor; C1, complement component 1 (C1q, C1r, C1s sub-units); C1inh, C1 inhibitor; C2, complement component 2 (and its split products C2a, C2b); C3, complement component 3 (and its split products C3a, C3b, C3c, C3d); C3aR, complement C3a receptor; C4, complement component 4 (and its split products C4a, C4b); C4BP, C4b-binding protein; C5, complement component 5; C5aR1, complement C5a receptor 1; C5aR2, complement C5a receptor 2; C9, complement component 9; CD, cluster of differentiation; CLR, collagen-like regions (of C1q); COVID-19, corona virus disease 2019; CP, complement classical pathway; CR1, complement receptor 1; CR2, complement receptor 2; CR3, complement receptor 3; CR4, complement receptor 4; DAF, decay acceleration factor; DAMPs, damage-associated molecular patterns; DNA, deoxyribonucleic acid; EDS, Ehlers-Danlos syndromes; ERK, extracellular signal-regulated kinases; FB, complement factor B (and its split products FBa, FBb); FD, complement factor D; FcγRIIIa, low affinity immunoglobulin gamma Fc region receptor III-A; FH, complement factor H; FI, complement factor I; GADPH, glyceraldehyde 3-phosphate deshydrogenase; gC1qR, globular C1q receptor; GR, globular regions (of C1q); GSPE, grape seed procyanidins extract; HLA-DR, human leukocyte antigen – DR isotype; HMGB1, high-mobility group box 1; ICs, Immune complexes; IFN, interferons; IFNγ, interferon gamma; IgG, immunoglobulin G; IgM, immunoglobulin M; IL-1β, interleukin 1 beta; IL-6, interleukin 6; ITIM, immune receptor tyrosine-based inhibitory motifs; JNK, c-Jun N-terminal kinase; LAIR-1, leukocyte-associated immunoglobulin-like receptor-1; LN, lupus nephritis; LP, complement lectin pathway; LPS, lipopolysaccharide; MASP-1, mannan-binding lectin serine protease 1; MASP-2, mannan-binding lectin serine protease 2; MD2, myeloid differentiation protein 2; MEK, Mitogen-activated protein kinase interleukine; miRNA, micro ribonucleic acid; MCP, membrane cofactor protein; MyD88, myeloid differentiation primary response 88; NF-κB, nuclear factor-kappa B; NK, natural killer; NLRP3, NOD-like receptor family pyrin domain containing 3; *P. gingivalis*, *Porphyromonas gingivalis*; PAMPs, pathogen-associated molecular patterns; pDC, plasmacytoid dendritic cells; pEDS, periodontal Ehlers-Danlos syndromes; PI3K, phosphoinositide 3-kinase; PKR, protein kinase R; RAGE, receptor for advanced glycation end products; SHP1, tyrosine phosphatase containing SH2 domain; SLE, systemic lupus erythematosus; SLEDAI, systemic lupus erythematosus disease activity index; SYK, spleen tyrosine kinase; TCR, T-cell receptor; TGF-β1, transforming growth factor beta 1; Th1, T helper cells 1; TLR2, toll-like receptor 2; TLR4, toll-like receptor 4; TLRs, toll-like receptors; TNF, tumor necrosis factors; TNFα, tumor necrosis factor alpha.

## Glossary

**Table:**

Abs	antibodies
ACAMPs	apoptotic cell-associated molecular patterns
Akt	protein kinase B
AMPs	associated molecular patterns
AP	complement alternative pathway
auto-Abs	autoantibodies
BAFF	B-cell activating factor
BCR	B-cell receptor
C1	complement component 1 (C1q, C1r, C1s sub-units)
C1inh	C1 inhibitor
C2	complement component 2 (and its split products C2a, C2b)
C3	complement component 3 (and its split products C3a, C3b, C3c, C3d)
C3aR	complement C3a receptor
C4	complement component 4 (and its split products C4a, C4b)
C4BP	C4b-binding protein
C5	complement component 5
C5aR1	complement C5a receptor 1
C5aR2	complement C5a receptor 2
C9	complement component 9
CD	cluster of differentiation
CLR	collagen-like regions (of C1q)
COVID-19	corona virus disease 2019
CP	complement classical pathway
CR1	complement receptor 1
CR2	complement receptor 2
CR3	complement receptor 3
CR4	complement receptor 4
DAF	decay accelerating factor
DAMPs	damage-associated molecular patterns
DNA	deoxyribonucleic acid
EDS	Ehlers-Danlos syndromes
ERK	extracellular signal-regulated kinases
FB	complement factor B (and its split products FBa, FBb)
FD	complement factor D
FcγRIIIa	low affinity immunoglobulin gamma Fc region receptor III-A
FH	complement factor H
FI	complement factor I
GADPH	glyceraldehyde 3-phosphate deshydrogenase
gC1qR	globular C1q receptor
GR	globular regions (of C1q)
GSPE	grape seed procyanidins extract
HLA-DR	human leukocyte antigen – DR isotype
HMGB1	high-mobility group box 1
ICs	Immune complexes
IFN	interferons
IFNγ	interferon gamma
IgG	immunoglobulin G
IgM	immunoglobulin M
IL-1β	interleukin 1 beta
IL-6	interleukin 6
ITIM	immune receptor tyrosine-based inhibitory motifs
JNK	c-Jun N-terminal kinase
LAIR-1	leukocyte-associated immunoglobulin-like receptor-1
LN	lupus nephritis
LP	complement lectin pathway
LPS	lipopolysaccharide
MASP-1	mannose-binding lectin associated serine protease 1
MASP-2	mannose-binding lectin associated serine protease 2
MD2	myeloid differentiation protein 2
MEK	Mitogen-activated protein kinase
miRNA	micro ribonucleic acid
MCP	membrane cofactor protein
MyD88	myeloid differentiation primary response 88
NF-κB	nuclear factor-kappa B
NK	natural killer
NLRP3	NOD-like receptor family pyrin domain containing 3
*P. gingivalis*	*Porphyromonas gingivalis*
PAMPs	pathogen-associated molecular patterns
pDC	plasmacytoid dendritic cells
pEDS	periodontal Ehlers-Danlos syndromes
PI3K	phosphoinositide 3-kinase
PKR	protein kinase R
RAGE	receptor for advanced glycation end products
SHP1	tyrosine phosphatase containing SH2 domain
SLE	systemic lupus erythematosus
SLEDAI	systemic lupus erythematosus disease activity index
SYK	spleen tyrosine kinase
TCR	T-cell receptor
TGF-β1	transforming growth factor beta 1
Th1	T helper cells 1
TLR2	toll-like receptor 2
TLR4	toll-like receptor 4
TLRs	toll-like receptors
TNF	tumor necrosis factors
TNFα	tumor necrosis factor alpha.
